# Exploring FTIR Spectroscopy as an Orthogonal Approach for Detecting Processing-Induced Changes in Extracellular Vesicle Preparations

**DOI:** 10.3390/ijms27219482

**Published:** 2026-10-23

**Authors:** Marija Tursunović, Lidija Filipović, Ninoslav Mitić, Milica Popović

**Affiliations:** 1 Innovative Centre of the Faculty of Chemistry, 11158 Belgrade, Serbia; 2 Institute for the Application of Nuclear Energy (INEP), University of Belgrade, 11080 Belgrade, Serbia; 3 Faculty of Chemistry, University of Belgrade, Studentski trg 12-16, 11158 Belgrade, Serbia

**Keywords:** extracellular vesicles, ATR-FTIR spectroscopy, extracellular vesicle characterization, label-free analysis, processing-induced alterations

## Abstract

Extracellular vesicles (EVs) require complementary methods for their comprehensive characterization. This study investigates the potential of attenuated total reflectance Fourier-transform infrared (ATR-FTIR) spectroscopy to provide orthogonal information on alterations in EV preparations subjected to different processing conditions. EVs were isolated using a previously established nanobody-based immunoaffinity approach, and further subjected to lyophilization, repeated freeze–thawing, or heat treatment followed by sonication. The EV preparations were characterized using nanoparticle tracking analysis, transmission electron microscopy, biochemical assays, flow cytometry and ATR-FTIR spectroscopy. Although the overall FTIR spectral profiles remained broadly similar, multivariate and targeted spectral analyses revealed differences in the heat-and-sonicated preparation, which also showed the most pronounced changes observed by complementary characterization methods. The findings provide proof-of-concept evidence that ATR-FTIR can provide orthogonal information for the assessment of processing-induced changes in EV preparations.

## 1. Introduction

Extracellular vesicles (EVs) are phospholipid bilayer-enclosed nanoparticles released by cells [1] and can be found in a wide range of biological fluids [2,3,4,5,6,7]. They have an important role in intercellular communication by transferring proteins, lipids, nucleic acids, and other bioactive molecules between cells [8]. Due to their distribution in biological fluids and their diverse molecular cargo, EVs have been recognized as potential non-invasive biomarkers [9,10], as well as promising therapeutic agents and drug delivery systems [11,12,13].

With the increasing translation of EV research into clinical and industrial applications, maintaining the quality, stability and comparability of EV preparations has become a crucial requirement [14]. However, processing and storage procedures—such as freezing, freeze–thaw cycles, lyophilization, and mechanical or thermal stress—may affect their physicochemical properties, resulting in changes to the particle integrity, molecular composition, or biological function [15,16,17]. Hence, robust analytical approaches—capable of detecting processing-induced changes—are required for quality control during EVs’ manufacturing, handling and storing.

The characterization of EV preparations is still challenging due to their complexity and heterogeneity [18]. Accordingly, the Minimal Information for Studies of Extracellular Vesicles (MISEV) guidelines [19] recommend the combined use of different complementary analytical approaches for the characterization of EV preparations. Particle size and concentration are usually determined using nanoparticle tracking analysis (NTA), dynamic light scattering (DLS) and nano-flow cytometry [20,21], while electron microscopy and atomic force microscopy provide information on particle morphology [22]. Molecular characterization relies on techniques such as Western blotting, flow cytometry, proteomics, lipidomics and nucleic acid analysis [23,24], allowing the identification of distinctive EV-associated biomolecules. Although each of these techniques provides important information, it assesses only specific aspects of EV preparation, highlighting the need for combined use of the multiple orthogonal approaches for comprehensive characterization.

Among the label-free analytical techniques, vibrational spectroscopic techniques such as Raman spectroscopy and Fourier-transform infrared spectroscopy (FTIR) have been employed for the characterization of complex biological samples [25,26]. These methods create characteristic spectral fingerprints that reflect molecular composition through the interaction of electromagnetic radiation with molecular vibrations in the sample [27]. In EV research, Raman spectroscopy has been applied for the characterization of EV preparations from different biological sources, as well as for the assessment of EV preparation purity and related functional properties [26,28,29]. The application of the FTIR spectroscopy, in particular, has been facilitated by the integration with the rapid sampling techniques, such as attenuated total reflectance (ATR) [25]. Due to its fast analysis, minimal required sample preparations, and its label-free nature, ATR-FTIR has emerged as an attractive tool for the characterization of EVs, where it has been mostly used for the biochemical characterization of isolated EV samples [30,31] and assessment of their purity [29], as well as the discrimination of EV populations according to their cellular origin and pathological state [30,32,33,34,35]. Despite these applications, to the best of our knowledge, only a few studies have explored the potential of ATR-FTIR for analyzing biochemical alterations associated with the processing and storage of EV preparations [36].

In this controlled proof-of-concept analytical study, we aimed to investigate whether ATR-FTIR spectroscopy could provide orthogonal information on alterations in plasma-derived EV preparations subjected to different processing conditions. A pooled EV preparation was divided into untreated, lyophilized, freeze–thawed, and heat-and-sonicated aliquots, with heat-and-sonicated included as an intentionally harsh disruptive treatment. Processing-induced changes were examined using complementary EV characterization methods together with ATR-FTIR to determine if the alterations were also reflected in the spectral fingerprints of the EV preparations. The study was designed as an initial analytical feasibility assessment to explore the potential of ATR-FTIR for detecting changes in EV preparations.

## 2. Results

### 2.1. Physicochemical and Morphological Characterization of EV Preparations

EV preparations were isolated from human plasma using the previously established nanobody-based immunoaffinity approach [37] and further subjected to the different processing conditions.

**Figure 1:**
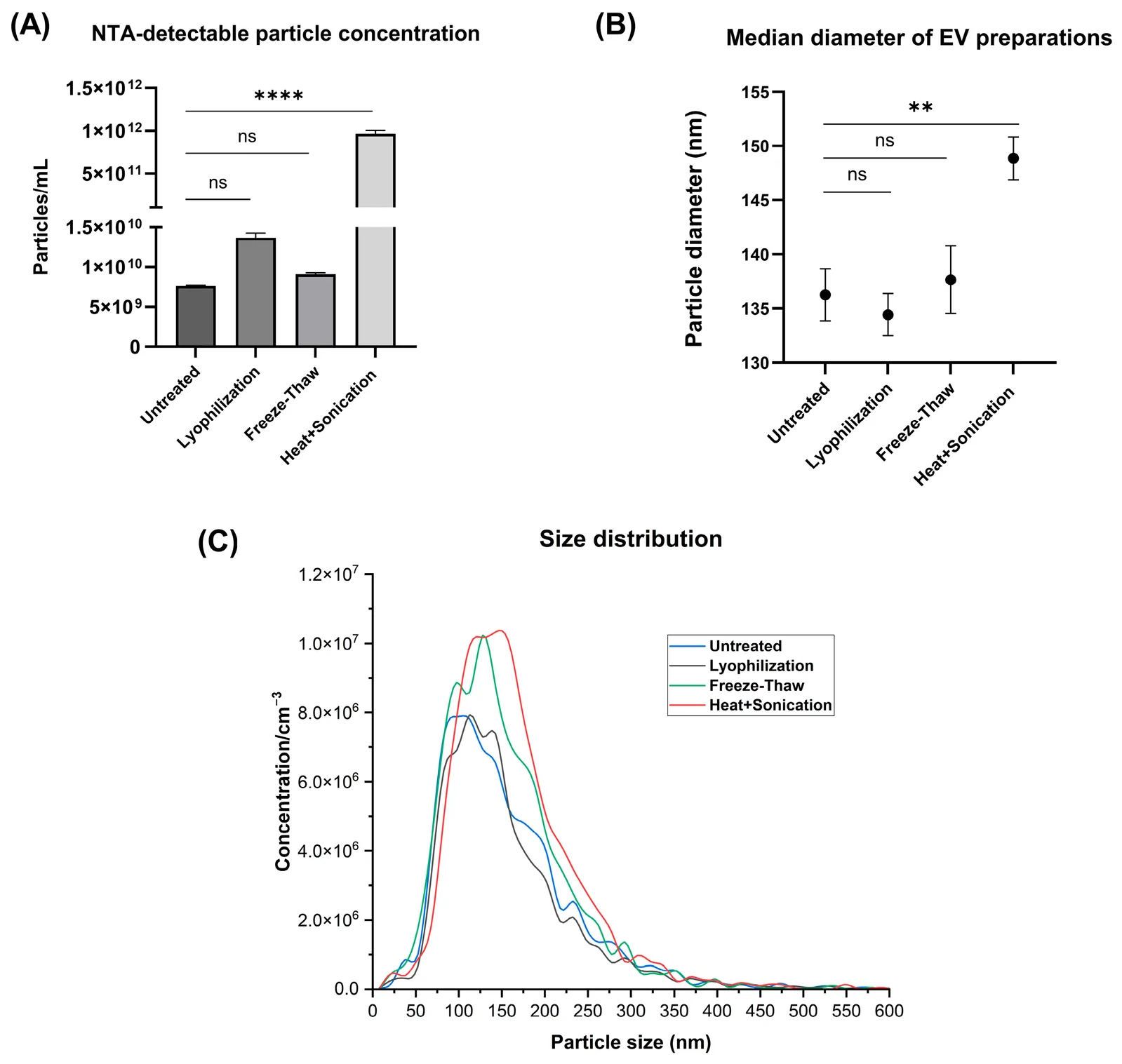
Effect of different processing methods on the physicochemical properties of EV preparations determined by NTA. (**A**) Particle concentration and (**B**) median diameter of differently processed EV preparations. (**C**) Representative particle size distribution for each experimental group. Statistical analysis was performed using one-way ANOVA followed by Dunnett’s multiple comparisons test against untreated EV preparation. Observed differences were considered statistically significant at *p* < 0.05. Statistical significance is indicated as ** *p* < 0.01, **** *p* < 0.0001, and ns, not significant (*p* ≥ 0.05). Data are presented as mean ± SD of technical replicates (*n* = 3). Statistical comparisons reflect technical measurement-level differences and should be interpreted as exploratory.

Nanoparticle tracking analysis (NTA) was performed for the evaluation of different processing methods on particle concentration and their size and distribution (Figure 1, Table 1). Heat-and-sonication was associated with an approximately 100 times increase in NTA-detectable particle concentration compared to untreated EV preparation, whereas lyophilization produced a slight increase in particle concentration. Freeze–thaw treatment showed a similar particle concentration compared to the untreated preparation. (Figure 1A). Heat-and-sonication treatment was also associated with an increase in the mean particle diameter from 136 nm to 149 nm, whereas no changes in particle size were observed following lyophilization or repeated freeze–thaw cycles (Figure 1B).

Following heat-and-sonication, the lower diameters side of the size distribution curve shifted slightly towards larger diameters (Figure 1C), consistent with an increase in D10 from 76.7 to 90.1 nm. Compared with the untreated preparation, the heat-and-sonicated preparation showed a lower share of detected particles smaller than 150 nm (49% vs. 56%), whereas the proportion of particles smaller than 200 nm remained relatively similar (74% vs. 76%). Notably, the heat-and-sonicated preparation also showed a higher proportion of particles in the 150–200 nm range (26%) compared with the untreated (19%), lyophilized (19%), and freeze–thawed (22%) preparations (Table 1). D90 also remained relatively unchanged, while span decreased from 1.3 to 1.1 (Table 1). Together, these findings indicate a modest narrowing of the measured particle size distribution in heat-and-sonicated preparation, primarily associated with changes at its lower-size end.

**Figure 2:**
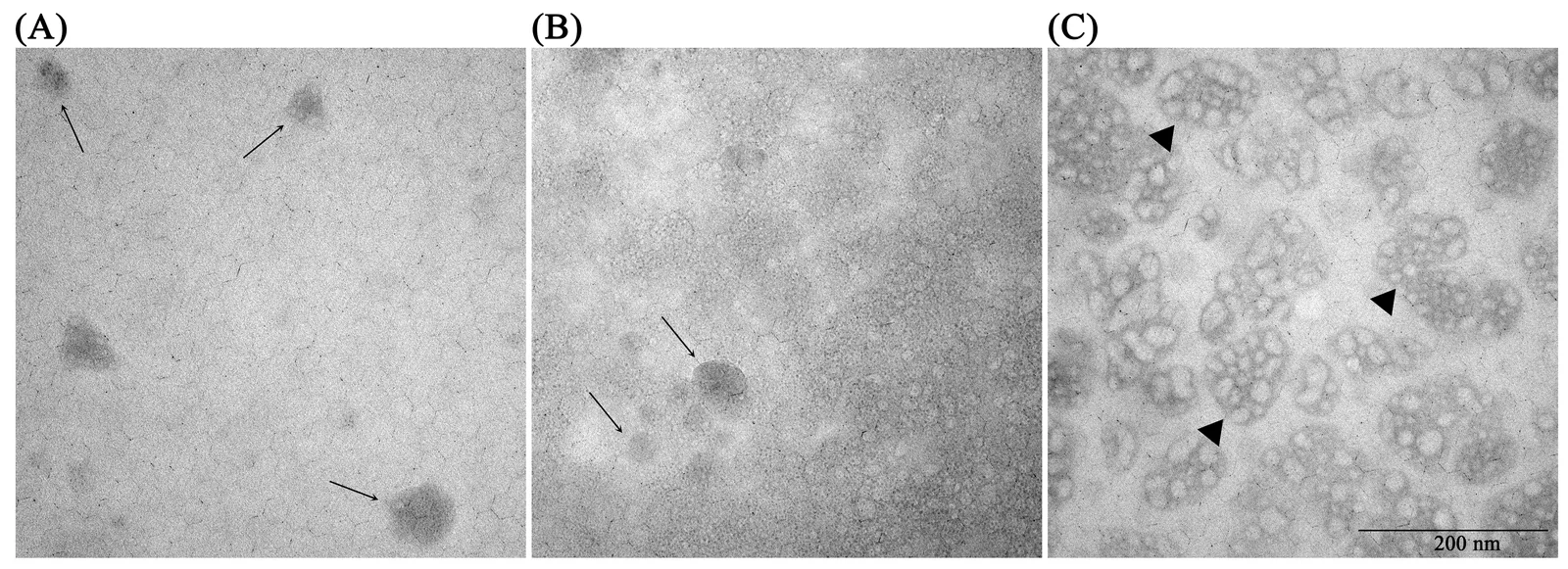
Representative transmission electron microscopy (TEM) images showing the size and structural heterogeneity of plasma EV preparations. (**A**) Untreated EV preparation. (**B**) EV preparation subjected to repeated freeze–thaw cycles. (**C**) EV preparation subjected to combined heat-and-sonication treatment. Scale bar = 200 nm. Arrows indicate round vesicle-like structures, while triangles indicate larger irregular clustered structures observed in the heat-and-sonicated preparation.

Transmission electron microscopy (TEM) was used for the morphological characterization of three representative EV preparations—the untreated preparation as a control; the heat-and-sonicated preparation, which showed the most pronounced changes across complementary methods; and the freeze–thawed preparation, which was selected to represent a processing condition associated with limited changes. Untreated and freeze–thawed preparations showed mostly rounded vesicle-like structures with broadly similar morphology. In contrast, the heat-and-sonicated preparation displayed greater morphological heterogeneity, including larger irregular, clustered structures in addition to smaller vesicle-like particles (Figure 2).

### 2.2. Biochemical Characterization of EV Preparations

**Figure 3:**
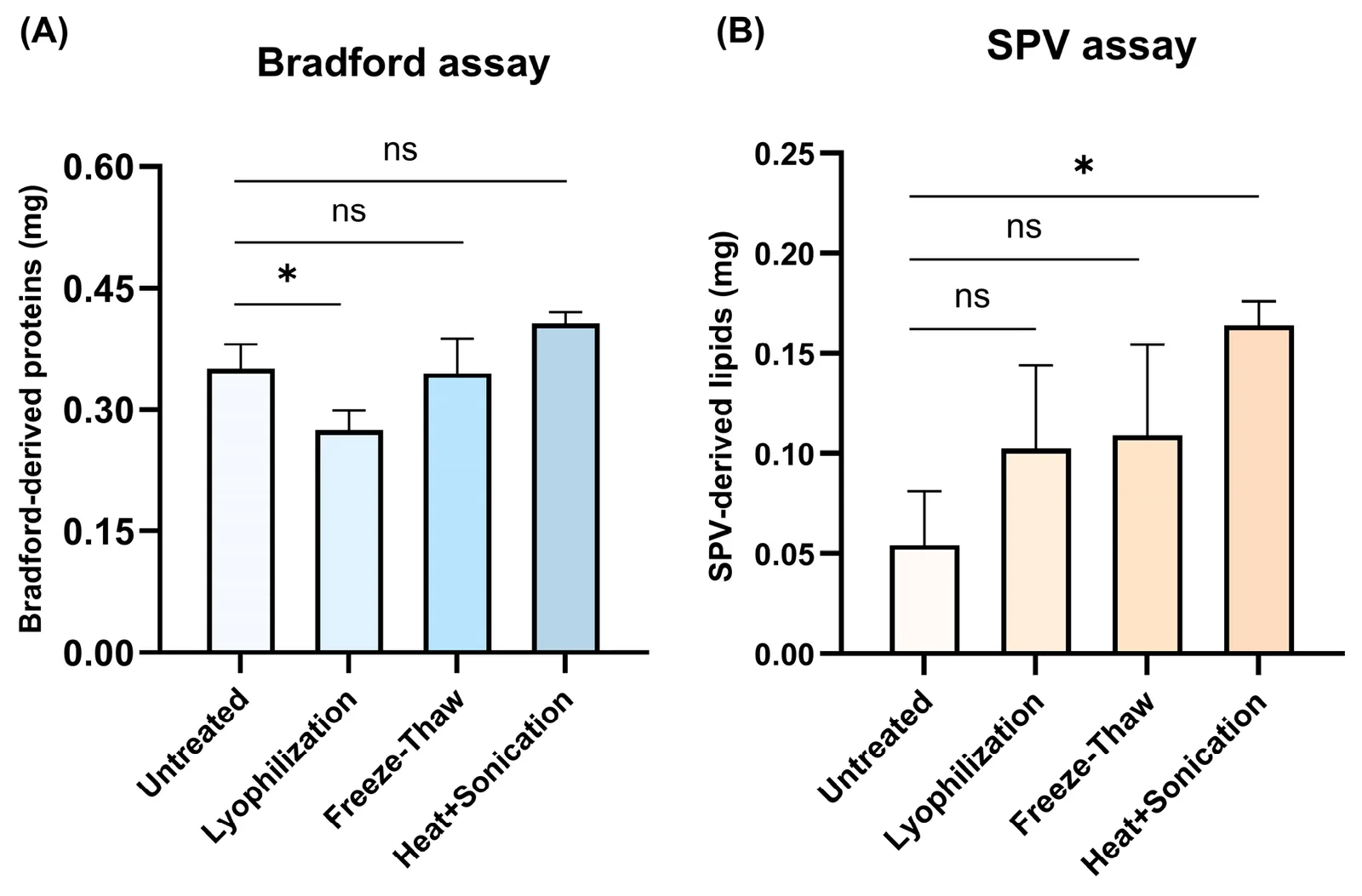
Bradford-derived protein and SPV-derived lipid measurements using colorimetric assays. (**A**) Bradford-derived protein measurement in untreated and processed EV preparations. (**B**) SPV-derived lipid signal in untreated and processed EV preparations. Statistical analysis was performed using one-way ANOVA followed by Dunnett’s multiple comparisons test. Statistical significance was defined as *p* < 0.05. Statistical significance is indicated as * *p* < 0.05 and ns, not significant (*p* ≥ 0.05). Data are presented as mean ± SD of technical replicates (*n* = 3). Statistical comparisons reflect technical measurement-level differences and should be interpreted as exploratory.

Proteins and lipids in each EV preparation were determined with Bradford and sulfo-phospho-vanillin (SPV) assays, respectively (Figure 3). Lyophilized preparation showed a lower Bradford-derived protein measurement, whereas freeze–thawed and heat-and-sonicated preparations showed similar values as the untreated one (Figure 3A). In contrast, heat-and-sonicated preparation had a higher SPV-derived signal than the untreated one, while smaller differences were observed for the other processing conditions (Figure 3B).

### 2.3. EV Surface Markers Analysis with Flow Cytometry

The flow cytometry analysis of common EV surface protein markers was performed by immobilization of EV preparations onto latex beads and further staining with fluorescently labeled monoclonal antibodies against CD9, CD63 and CD81. Equal protein amounts of each EV preparation were used for the coupling of latex beads. All samples produced a clear positive fluorescence shift relative to the autofluorescence of latex beads alone, confirming the presence of all three surface markers.

**Figure 4:**
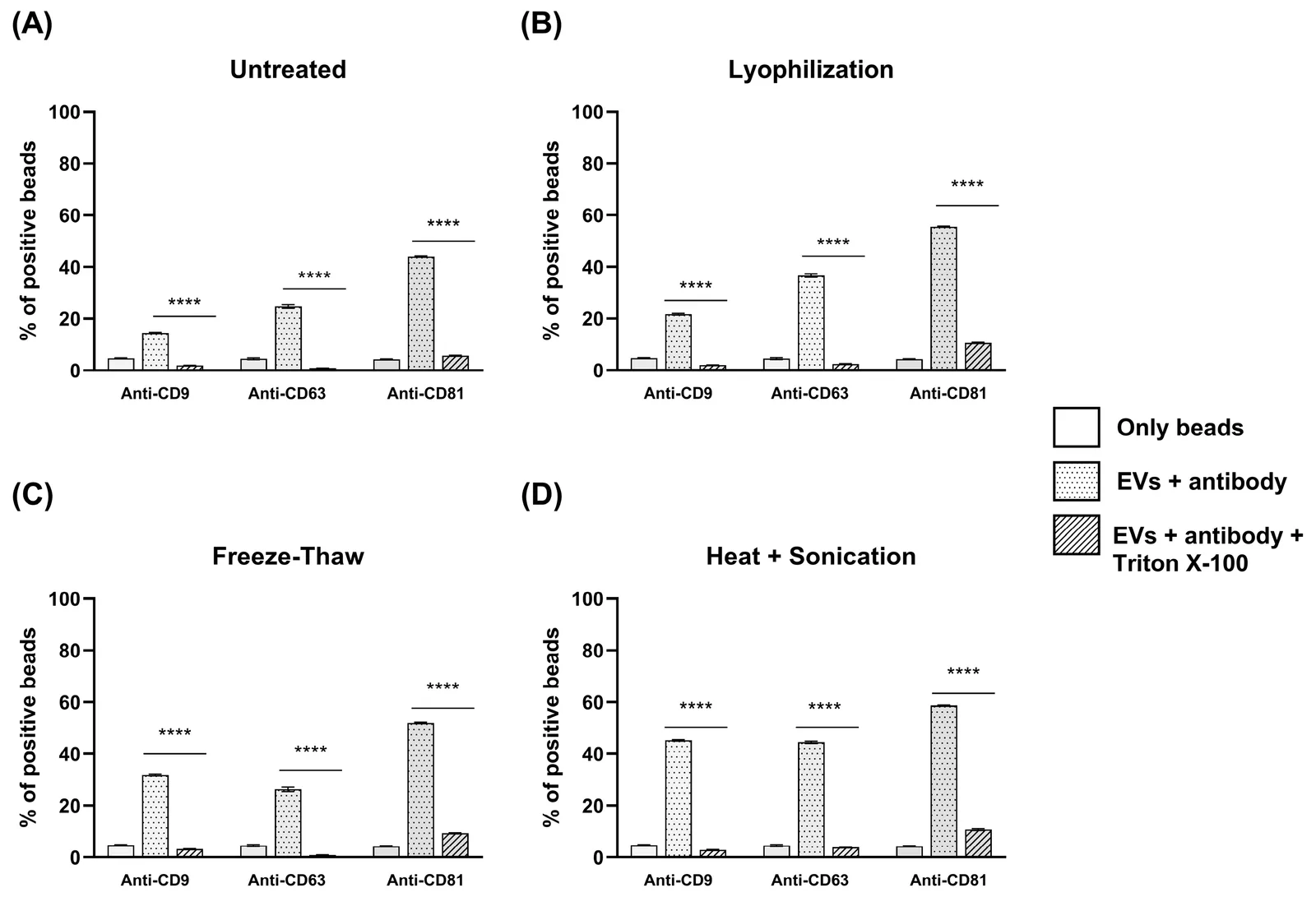
Detection of EV-associated surface markers using flow cytometry analysis. Representative histograms showing the detection of the common EV surface markers, CD9, CD63 and CD81 in (**A**) untreated, (**B**) lyophilized, (**C**) freeze–thawed, and (**D**) heat-and-sonicated EV preparations. Dotted histograms represent the fluorescence shifts obtained after staining with fluorescently labeled anti-CD9, anti-CD63 and anti-CD81 antibodies, while solid-line histograms represent the fluorescence shifts obtained after detergent (triton X-100) treatment. Detergent treatment was included to support that the detected fluorescence originated from lipid membrane structures. Statistical analysis was performed between EV preparations before and after detergent treatment within each processing condition using one-way ANOVA followed by Tukey’s multiple comparisons test. Differences were considered statistically significant at *p* < 0.05. Statistical significance is indicated as **** *p* < 0.0001. Data are presented as mean ± SD of technical replicates (*n* = 3). Statistical comparisons reflect technical measurement-level differences and should be interpreted as exploratory. Comparisons between processing groups were interpreted semi quantitatively and were not subjected to statistical analysis.

However, differences in the proportion of tetraspanin-positive beads were observed among the processing conditions. In the untreated sample, 14.6%, 24.7%, and 44% of beads were positive for CD9, CD63, and CD81, respectively. After lyophilization, the proportions of positive beads were 21.6%, 36.6%, and 55.6%, while freeze–thaw treatment resulted in 31.9%, 26.3%, and 51.9% positive beads for CD9, CD63, and CD81, respectively. Heat-and-sonication processing produced the highest apparent marker detection, with 45.3%, 44.5%, and 58.7% of beads staining positive for CD9, CD63, and CD81, respectively (Figure 4).

Detergent treatment significantly reduced the percentage of positive beads in all samples for all three surface markers, supporting that detected fluorescence shifts were associated with lipid membrane structures.

### 2.4. Molecular Characterization of EV Preparations by ATR-FTIR Spectroscopy

#### 2.4.1. ATR-FTIR Spectral Analysis

**Figure 5:**
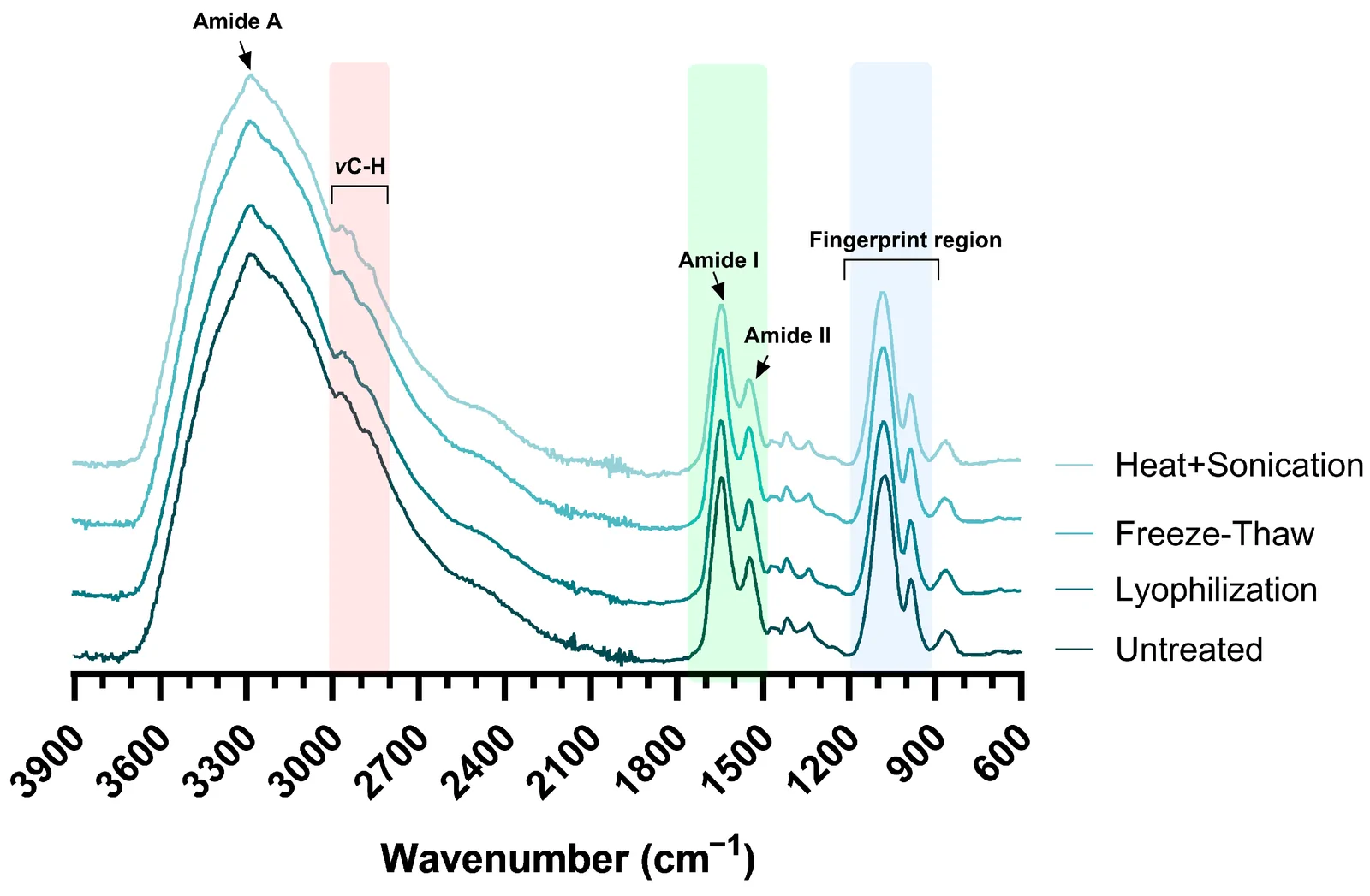
Representative ATR-FTIR spectra of untreated and differently processed EV preparations. Spectra represent the mean of nine acquired spectra per each group. Shaded regions correspond to the C-H stretching region (red), the protein amide I and amide II regions (green), and the fingerprint region (blue).

FTIR spectra of untreated and processed EV preparations are shown in Figure 5. All four preparations have highly similar spectral profiles, with prominent absorption bands commonly found in extracellular vesicle samples [30].

A wide band between ~3800 and ~3000 cm^−1^ is assigned to overlapping O-H and N-H stretching vibrations predominantly originating from carbohydrates and proteins [38]. The C-H stretching region (~2985–2835 cm^−1^) is commonly associated with the symmetric and asymmetric stretching vibrations of CH_2_ and CH_3_ groups present in membrane lipids [39].

The protein region is dominated by the amide I and amide II bands, originating from the C=O stretching and N–H bending/C–N stretching vibrations of the peptide bonds [40]. Additionally, the spectra exhibit the amide A band (~3300 cm^−1^) originating from N-H stretching vibrations of the peptide bonds [40].

The fingerprint region (~1300–900 cm^−1^) contains absorption bands originating from phospholipids, carbohydrates and nucleic acid [41].

**Figure 6:**
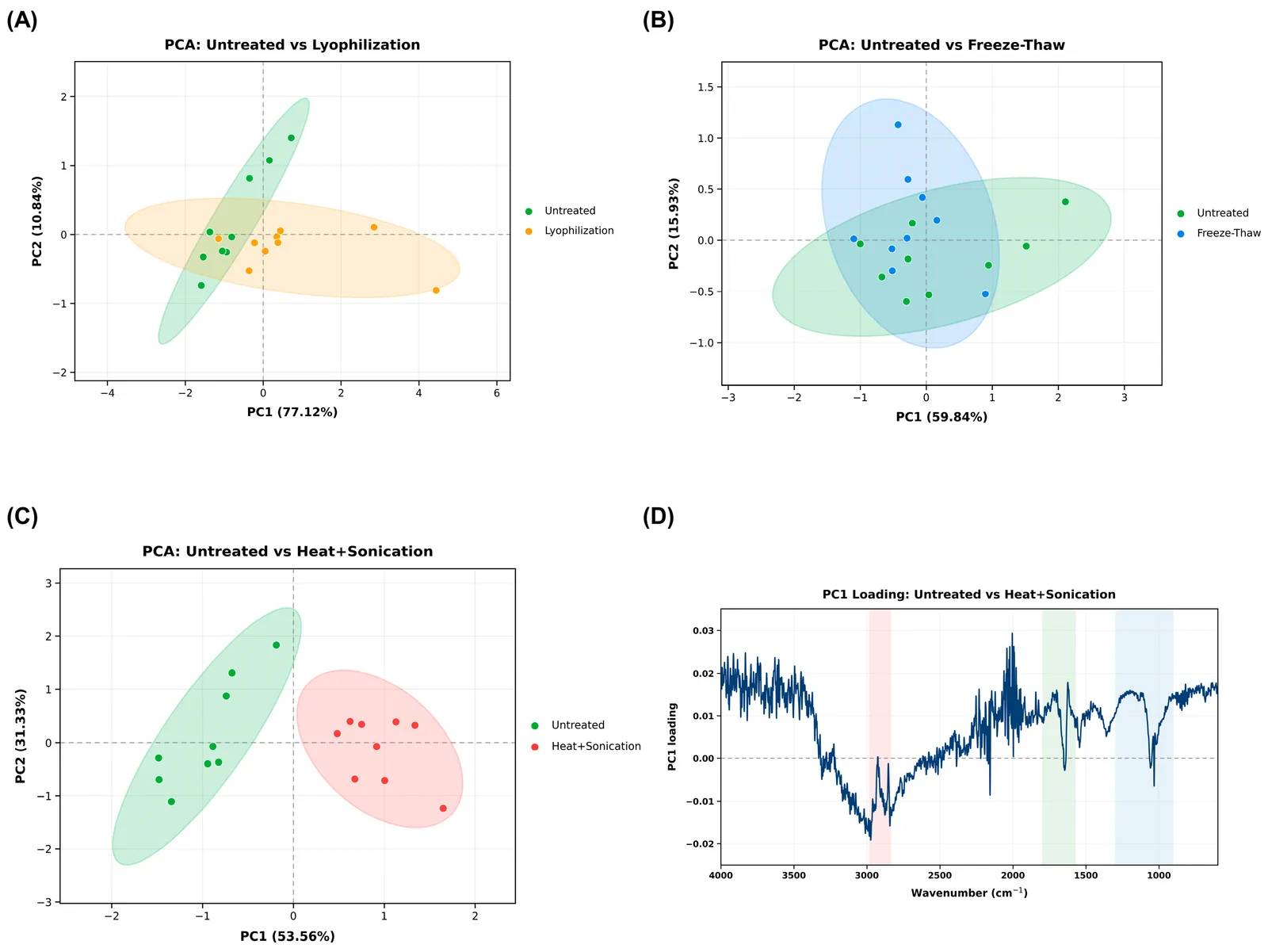
Pairwise PCA of FTIR spectra and PC1 loading plot. PCA score plots comparing untreated EV preparation with (**A**) lyophilized, (**B**) freeze–thawed and (**C**) heat-and-sonicated EV preparations, with 95% confidence ellipses. The percentages on the axes correspond to the variance explained by PC1 and PC2. Each point represents one spectrum (*n* = 9 spectra per processing-treatment) (**D**) PC1 loading plot for the untreated versus heat and sonicated groups, which showed the clearest group separation. Shaded regions indicate regions typically associated with lipids (red), proteins (green) and carbohydrates/nucleic acids (blue).

Overall, the spectra profiles seemed mostly conserved across all processing conditions, with no obvious differences between all four EV preparations. Therefore, principal component analysis (PCA) was performed to determine whether subtle spectral alterations could be identified with multivariate analysis (Figure 6). Pairwise PCA comparison of untreated EV preparations with each processing method demonstrated varying degrees of discrimination between each group. The clearest separation was observed between the untreated and the heat-and-sonicated groups, suggesting that this type of processing produced the greatest alteration in their spectral profile (Figure 6C). In contrast, comparison of the untreated group with the lyophilized (Figure 6A) and freeze–thawed EV preparations (Figure 6B) showed greater overlap between the groups. The first two principal components explained 87.96%, 75.77% and 84.89% of the total variance for the untreated versus lyophilized, untreated versus freeze–thawed and untreated versus heat-and-sonicated groups, respectively. Pairwise PCA comparisons among processed EV preparations are presented in Supplementary Figure S1. Since the highest separation was obtained between untreated and heat-and-sonicated preparations, PC1 loading analysis was performed for this comparison to examine the spectral regions contributing to the observed separation (Figure 6D). The PC1 loading profile showed prominent features in the C-H stretching, amide I and II regions associated with proteins, and the fingerprint region, indicating that spectral variations from these domains contributed to the separation between these two groups. Additional PCA of untreated and heat-and-sonicated preparations was performed using the C–H stretching region (3000–2800 cm^−1^), the Amide I/II region (1700–1500 cm^−1^), and their concatenation. Separation between the two preparations was observed in all three analyses, although some overlap remained in the C–H stretching region (Supplementary Figure S2).

**Figure 7:**
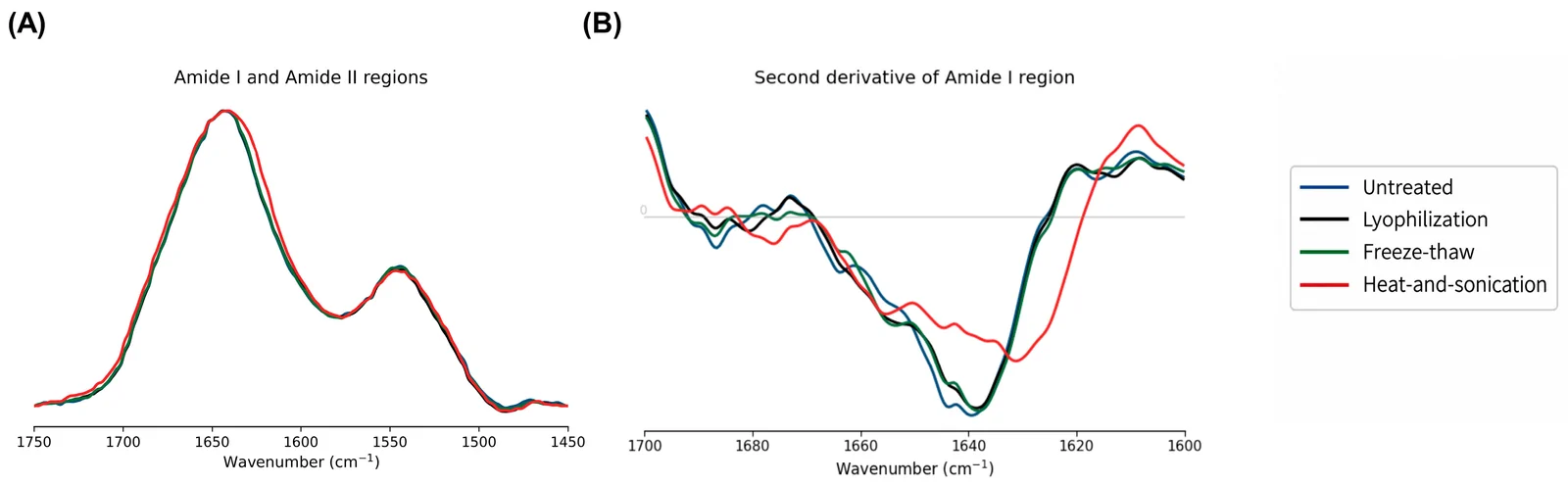
Detailed comparison of protein-associated ATR-FTIR spectral regions in untreated and processed EV preparations. (**A**) Overlaid Amide I and Amide II spectral profiles. (**B**) Second-derivative spectra of the Amide I region. Spectra represent the mean of nine technical measurements per processing condition.

To further examine processing-associated differences in protein-related spectral features, the shapes of the Amide I and Amide II bands were compared (Figure 7A). The spectra showed broadly similar profiles across all four EV preparations. However, the heat-and-sonicated preparation exhibited a slightly broader and flatter Amide I band, with slight differences on the lower-wavenumber side, whereas the Amide II profiles remained largely comparable among the preparations.

To further resolve subtle differences within the Amide I region, second-derivative analysis was performed (Figure 7B). The heat-and-sonicated preparation showed the most pronounced differences relative to untreated EVs, while the lyophilized and freeze–thawed preparations retained more similar derivative profiles.

#### 2.4.2. Comparison of Colorimetric and Spectroscopic Protein-to-Lipid Ratios

**Figure 8:**
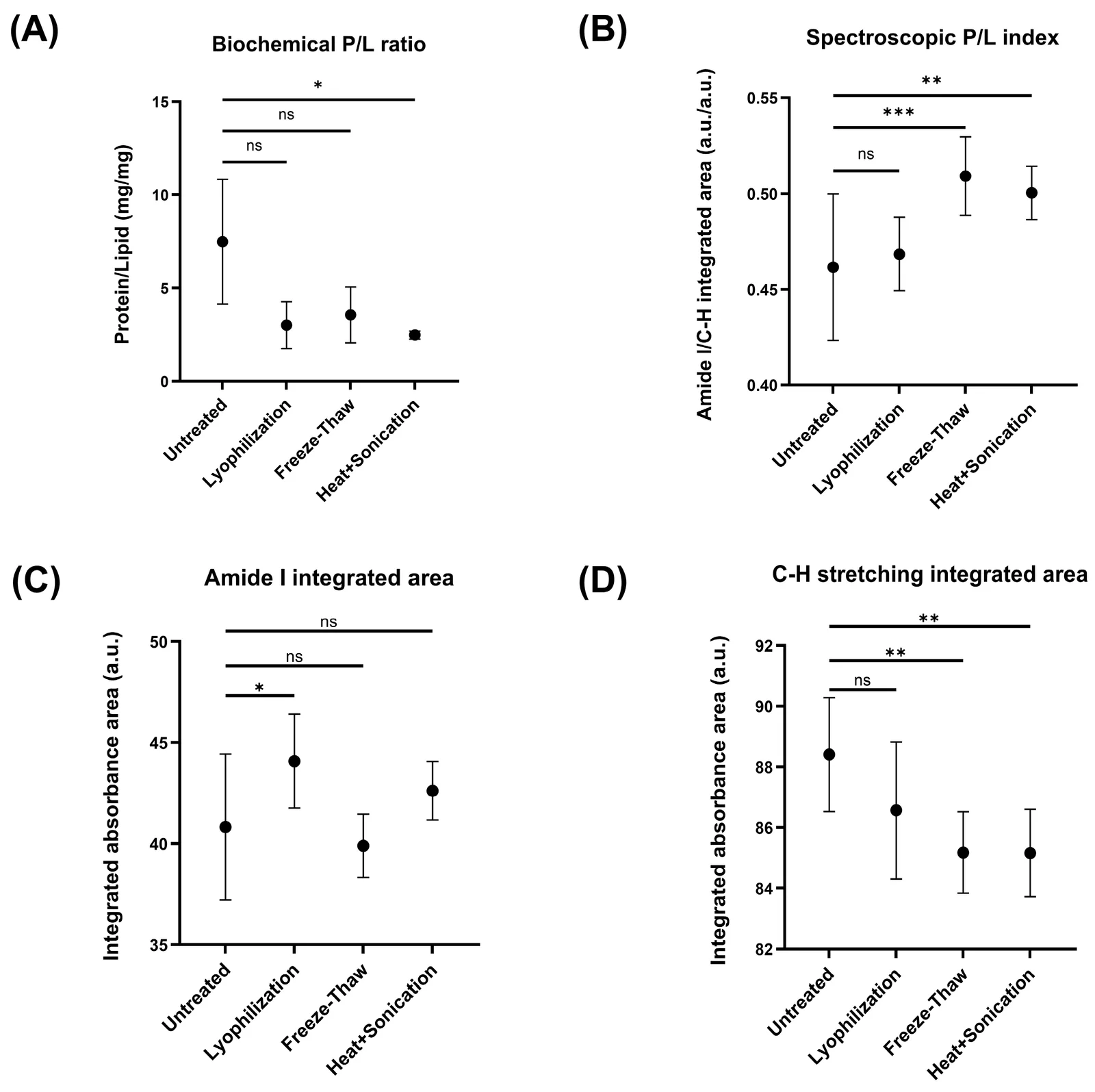
Comparison of biochemical and spectroscopic protein-to-lipid measurements and integrated FTIR band areas in untreated and processed EV preparations. (**A**) Biochemical P/L ratio calculated from Bradford-derived protein and SPV-derived lipid measurements, using BSA and cholesterol as calibration standards, respectively. (**B**) Spectroscopic P/L index, defined as the ratio of the integrated Amide I band area to the integrated C–H stretching band area. (**C**) Integrated absorbance area of the Amide I band. (**D**) Integrated absorbance area of the C–H stretching region. The biochemical P/L ratio is a mass-based ratio expressed as mg/mg, whereas the spectroscopic P/L index is a dimensionless ratio of integrated spectral areas; the two measures are not directly equivalent.” Statistical analysis was performed using one-way ANOVA followed by Dunnett’s multiple-comparisons test. Statistical significance was defined as *p* < 0.05. Statistical significance is indicated as * *p* < 0.05, ** *p* < 0.01, *** *p* < 0.001, and ns, not significant (*p* ≥ 0.05). Data are presented as mean ± SD of technical replicates. Statistical comparisons reflect technical measurement-level differences and should be interpreted as exploratory.

To compare the protein-to-lipid relationships obtained using two different analytical approaches, a biochemical P/L ratio and FTIR-derived spectroscopic P/L index were calculated for each EV preparation (Figure 8A,B; Supplementary Table S1). The biochemical P/L ratio was calculated from the Bradford-derived protein and SPV-derived lipid measured signals, using BSA and cholesterol as calibration standards. Spectroscopic P/L index was defined as the ratio of the integrated Amide I band area to the integrated C-H stretching band area (Section 4.5). The spectroscopic P/L index reflects the relative contributions of these two spectral regions and should not be interpreted as a direct measure of protein-to-lipid mass composition.

The biochemical P/L ratio was highest in the untreated EV preparation and decreased upon processing methods, with the largest reduction observed after heat-and-sonication (Figure 8A). In contrast, the spectroscopic P/L index showed minor changes among processing conditions. Untreated EV preparation had the lowest index, while higher indices were observed after freeze–thaw and heat-and-sonication treatment (Figure 8B). Although both analytical approaches detected processing-dependent changes, the biochemical P/L ratio and spectroscopic P/L index exhibited opposite trends across the treatment groups.

To investigate spectral contributions underlying these changes, the integrated absorbance areas of the amide I band and the C-H stretching region were analyzed separately (Figure 8C,D). The integrated amide I area had a modest increase upon lyophilization compared to untreated EV preparation, while no significant differences were noticed for other two processing conditions (Figure 8C). Conversely, the integrated C-H stretching area showed a decreasing trend, and this integrated region was lower in the freeze–thawed and heat-and-sonicated preparations (Figure 8D).

## 3. Discussion

Through the integration of complementary analytical techniques, this study examined the effects of different processing conditions within a common plasma-derived EV preparation. In addition, the study investigated whether these changes were also reflected in the ATR-FTIR spectral fingerprints, thereby assessing its potential to provide orthogonal analytical information alongside conventional EV characterization methods.

Among the processing methods investigated in this study, repeated freeze–thawing and lyophilization produced limited changes in the physicochemical properties of EV preparations. Similar observations were reported, where a single freeze–thaw cycle had minimal effects on plasma-derived EV concentration and size [42]. Although their experimental design differed from ours, in which EV preparation was subjected to ten freeze–thaw cycles, in both studies NTA did not detect any significant changes regarding their concentration and diameter under freezing conditions. In contrast, other studies reported a progressive reduction in EV recovery, with an increase in particle size upon multiple freeze–thaw cycles, indicating membrane damage and vesicle aggregation [43]. Similarly, lyophilization did not significantly affect particle concentration or mean particle diameter in the present study, although in the literature it has been reported that lyophilization in the absence of cryo- or lypo-protectants resulted in significantly lower particle concentration, while particle size remained mainly unchanged [44]. Together, these findings indicate that the response of EVs to freeze–thawing and lyophilization is not universal and may be influenced by multiple factors, including different EV sources, plasma processing, and isolation methodology, as well as experimental and analytical protocols [16,19,45,46].

Unlike freeze–thawing and lyophilization, heat-and-sonication was included in this study as an intentionally harsher disruptive processing method to evaluate the ability of the analytical workflow to detect pronounced alterations in EV preparations. Notably, membrane-disruptive approaches such as freeze–thaw cycling and sonication have also been used for exogenous cargo loading into EVs, highlighting the relevance of assessing their effects on EV physicochemical and molecular properties [47,48]. In the present study, heat-and-sonication treatment was associated with approximately 100 times higher NTA-detectable particle concentration, together with an increase in particle diameter upon this treatment. Additional analyses of the size distribution parameters indicated that the shift towards larger particle diameters was most evident at the lower-diameter end of the distribution, as well as in 150–200 nm particle diameter range, with a modest overall narrowing in the heat-and-sonicated preparation (Table 1). An increase in NTA-measured particle diameter in EV preparations upon sonication has been previously reported in sonication-based EV loading studies [47,48]. Haney et al. observed an increase in the NTA-measured diameter of sonicated EVs and attributed this change to the substantial reformation and reshaping of exosomal membranes [47]. Similarly, Kim et al. reported larger NTA-measured particle size of EVs after sonication and likewise suggested that sonication may promote membrane reorganization [48]. Sonication may also affect the number of particles detected by NTA. Recently, it has been reported that mild water bath sonication significantly increased the measurable particle concentration, which was explained through disaggregation of EVs and therefore an increased particle recovery during NTA [49]. However, in the present study, considerably harsher thermal and mechanical conditions were applied. The magnitude of the increase reported in that study was considerably smaller than the approximately 100-fold difference observed here, suggesting that disaggregation alone is unlikely to account for the present result.

The obtained increase in both NTA-detectable particle concentration and particle diameter therefore suggests that heat-and-sonication harsh treatment considerably altered the physical organization of the EV preparation. This may be a result of several processes occurring simultaneously, such as the disruption of EV membranes, formation of membrane fragments, aggregation, disaggregation of pre-existing clusters, and reorganization of lipid components into new membrane-associated assemblies. Since NTA cannot distinguish intact EVs from other particles with similar size [50], the exact structural identity of the NTA-detected particles in heat-and-sonicated cannot be determined by NTA alone. TEM analysis of representative untreated, freeze–thawed, and heat-and-sonicated preparations revealed however greater morphological differences in the heat-and-sonicated preparation, including larger irregular clustered structures together with smaller vesicle-like particles. The presence of such structures may reflect membrane reformation and reshaping upon sonication, as previously suggested [47,48]. In addition, considering the higher SPV-derived lipid signal (Figure 3B) and higher apparent detection of membrane associated tetraspanins upon heat-and-sonication (Figure 4), it is plausible that such structural changes could have influenced lipid accessibility in the colorimetric assay and the accessibility of tetraspanin epitopes during flow cytometry analysis.

Flow cytometry showed that the highest apparent detection of all three tetraspanins has been observed after heat-and-sonication, while modest increases were also observed upon lyophilization and freeze–thaw cycles. However, bead-based flow cytometry provides only a semiquantitative assessment of EV surface markers, where fluorescent signal represents the cumulative detection of multiple vesicles captured on individual beads [51]. The measured signal can therefore be influenced by several factors, including the number of particles captured on individual beads, their interaction with the bead surface, and the accessibility or presentation of membrane-associated epitopes. Accordingly, the increased apparent detection of membrane surface markers may reflect changes in all of these parameters. Detergent treatment markedly reduced fluorescence signal in all processing groups, supporting that the detected tetraspanin signal originated mostly from membrane-associated structures instead of non-membranous protein aggregates [52].

After assessment of the physicochemical, morphological and molecular effects on the investigated processing methods by conventional characterization techniques, ATR-FTIR was further examined as an orthogonal method for detecting processing-associated changes in the same EV preparations. ATR-FTIR has recently emerged as a promising analytical technique for EV characterization due to its rapid, label-free nature and its ability to provide a comprehensive biochemical fingerprint of the EV preparations using minimal sample preparation and small sample volumes [53]. Accordingly, it has been proposed for the characterization and quality control of EV preparations through the evaluation of spectral profiles and spectroscopic protein-to-lipid ratios [29,30,34,38,53]. However, despite growing interest in this technique, to the best of our knowledge, only a limited number of studies have investigated its ability to detect molecular alterations induced by different processing methods [36].

Representative ATR-FTIR spectra in this study showed comparable spectral profiles for all processing conditions. This is not surprising since EV spectra contain many overlapping bands from proteins, lipids, nucleic acids, and carbohydrates, which may make it hard to identify subtle molecular differences visually. Therefore, multivariate statistical analysis is widely used to reveal hidden spectral fluctuations [34,41,54]. In the present study, PCA of lyophilized and freeze–thawed EV preparations showed considerable overlap, whereas heat-and-sonication formed a distinct cluster in comparison to the untreated sample. Similar separation of the heat-and-sonicated group from other processed groups was also observed in pairwise PCA comparison (Supplementary Figure S1), indicating that this processing method introduced detectable changes in the molecular fingerprint of the EV preparations. Because the EV preparations were measured after drying from PBS and a matched dried PBS spectrum was not available, a contribution of residual buffer components to the fingerprint region cannot be completely excluded. Accordingly, spectral features within this region were interpreted cautiously and were not assigned exclusively to EV-derived molecular components. In addition, the observed changes in the Amide I band shape and second derivative profiles indicate that the harsh processing condition affected, to a certain extent, protein-related spectral features. Given the well-established sensitivity of the Amide I region to protein backbone conformation and secondary structure [40], this region may be particularly informative for the further investigation of processing-related protein structural changes. Future studies should examine these changes in greater detail across independent EV preparations to determine if they correspond to specific protein secondary-structure changes and to establish which Amide I-derived parameters could provide sufficient information for detecting different degrees of processing-associated changes in EV preparations.

Interestingly, the biochemical protein-to-lipid ratios and spectroscopic protein-to-lipid indices showed opposite trends after processing. These opposite trends most likely originate from different analytical principles of the two approaches and should not be considered directly equivalent. Bradford and SPV assays detect proteins and lipids through reagent-based chemical reactions with the analytes [55,56]. Therefore, the measured amounts may be influenced by the accessibility of these biomolecules to the assay reagents and should not be interpreted directly as corresponding changes in absolute protein or lipid mass. In contrast, the ATR-FTIR-derived P/L index is calculated from the integrated areas of the amide I and C-H stretching bands. Hence, the spectroscopic P/L index is a dimensionless parameter that reflects the relative spectroscopic contributions of these spectral regions. In the present study, no calibration between the integrated spectral areas and protein or lipid mass was established, therefore the spectroscopic P/L index should be interpreted as a relative spectral parameter, rather than a direct biochemical protein-to-lipid mass ratio. In addition, because the integrated spectral parameters were derived from spectra subjected to scale normalization, their absolute values may depend on the applied preprocessing procedure and normalization range. Therefore, the integrated band areas and the derived spectroscopic P/L index should be interpreted only as relative parameters for comparison among spectra processed under identical conditions.

Integrated band analysis revealed that the increased spectroscopic P/L index was predominantly a result of a reduced relative contribution of C-H stretching region upon processing, while the integrated amide I region remained largely unchanged (Figure 8C,D). The C–H stretching region has been widely used in FTIR studies of EVs as a lipid-associated spectral region because of the strong contribution of CH_2_ and CH_3_ stretching vibrations from membrane lipid acyl chains [30,38]. Lipid-associated C-H stretching bands may be influenced not only by lipid abundance, but also by the conformational state and packing of lipid acyl chains [57]. Heat-and-sonication could alter membrane packing or organization and modify the spectral contribution of lipid-associated hydrocarbon chains while simultaneously changing the accessibility of lipid-containing material to the SPV reagent. However, interpretation of C-H stretching region should remain cautious, as these vibrations are not exclusively derived from lipids and may also reflect contributions from other biomolecular components.

Taken together, several limitations should be considered during the interpretation of these findings. All processing conditions were applied to aliquots derived from a single pooled EV preparation; therefore, they do not illustrate biological variability between independent EV preparations. The design of the study was a proof-of-concept comparison within the same starting EV preparation, which is why the observed processing-induced differences should not be generalized across EV sources or isolation workflows. In addition, heat-and-sonication was intentionally selected as a severe disruptive condition and does not represent routine EV storage or handling. Under the present experimental conditions, spectral differences were observed for the processing method that exhibited the most pronounced changes using complementary EV characterization methods. However, the ability of ATR-FTIR to detect more subtle processing-induced changes remains to be established. Therefore, future studies should include independent EV preparations, additional processing treatments, and predefined analytical performance criteria to establish the reproducibility, sensitivity, and broader applicability of ATR-FTIR for EV quality assessment.

## 4. Materials and Methods

### 4.1. Purification of EV from Human Plasma

Extracellular vesicles are isolated from a healthy donor human plasma using a previously reported nanobody-based immunoaffinity approach [37]. Three nanobody-eGFP constructs labeled as NA8, ND10_1_ and ND10_2_ were used. Production and purification of the constructs, as well as human plasma collection and EV isolation, were done as previously described with some minor modifications [37]. Nanobodies were coupled to the polymer by incubating 250 μL of wet polymer with 80 μg of each nanobody. Immobilized polymer (250 μL) was incubated with 900 μL of 3 x diluted plasma with PBS for 1 h at RT with light rotating. EVs are further purified as already described [37]. Four separate isolations were performed to obtain sufficient material for the following analytical workflow. The resulting EV preparations were dialyzed against 0.2 μm filtered PBS.

### 4.2. Processing of EV Preparations

After dialysis, the four EV isolates were pooled into one fraction to generate a common starting EV preparation for comparison across processing conditions. Pooling was used so that all processing conditions could be applied to aliquots derived from the same starting matrix, thereby avoiding additional between-isolation variability in the treatment comparison. The pool of EV preparation was further divided into four aliquots corresponding to the untreated, lyophilized, freeze–thawed, and heat-and-sonicated conditions.

One aliquot was divided into smaller aliquots, frozen at −80 °C and lyophilized using a Martin Christ Alpha 2–4 LD plus freeze-dryer (Martin Christ Gefriertrocknungsanlagen GmbH, Osterode am Harz, Germany) at a chamber pressure of 0.080 mbar for 17 h (ice condenser temperature: −85 °C). Prior to analysis, the lyophilized EV samples were reconstituted with Milli-Q water to the original starting volume.

The second aliquot was subjected to repeated freeze–thaw cycles. EV preparation was frozen at −80 °C, then thawed in lukewarm water. This process was repeated 10 times.

The third aliquot was heated at 95 °C for 15 min and then sonicated using a BANDELIN SONOPULS HD 2070 ultrasonic homogenizer (BANDELIN electronic GmbH & Co. KG, Berlin, Germany; 20 kHz). Sonication was performed for 5 min at 50% amplitude using a pulsed regime of 20 s on/40 s off.

The fourth aliquot was left untreated and served as the reference preparation for all processing conditions. All four aliquots were further subdivided into smaller aliquots to avoid repeated freeze–thaw cycles and stored at −80 °C until further analysis.

### 4.3. Determination of Protein and Lipid Concentration in EV Preparations

Protein concentration was calculated with the Bradford assay [56] by mixing 200 μL of work reagent with 5 μL of the sample. BSA was used as a standard, and the absorbance was measured at 595 nm.

Sulfo-phospho-vanillin (SPV) assay was used as previously described for the determination of lipid concentration [57], using cholesterol as the calibration standard. The resulting SPV-derived lipid values are therefore expressed as cholesterol equivalents rather than absolute total lipid concentrations.

### 4.4. Nanoparticle Tracking Analysis (NTA) of EV Preparations

The size distribution and median diameter of the EV preparations were evaluated with a ZetaView Quatt PMX-430 (Particle Metrix, Inning am Ammersee, Germany) nanoparticle tracking analyzer and ZetaView software version 8.05.16. Before the analysis, the camera and laser were subjected to an automatic cell verification, with focus optimization using 100 nm polystyrene beads as outlined in the manufacturer’s instructions. EV preparations were diluted in particle-free 0.05 M PBS, pH 7.2 (dPBS) to achieve an optimal particle count per frame. Measurements were performed with a blue laser (488 nm) in light scatter mode, with washing steps with dPBS between each measurement. The video capture parameters comprised a shutter speed of 100 and a frame rate of 30 per cycle, with sensitivity adjusted to 78. The settings for post video capture were a minimum area of 10, a maximum area of 1000, and a minimum brightness of 30. Each EV isolate was assessed three times at up to 11 positions.

### 4.5. Attenuated Total Reflection Fourier-Transform Infrared Spectroscopy (ATR-FTIR)

Spectra were measured using Nicolet Summit FTIR Spectrometer (Thermo Fisher Scientific, Waltham, MA, USA) in ATR mode equipped with a DTGS KBr detector (Thermo Fisher Scientific, Waltham, MA, USA).

Aliquots (2 μL) of each EV preparation suspended in PBS were layered on a diamond crystal and dried under a gentle stream of nitrogen. Spectra (400–4000 cm^−1^) were collected in 32 scans with a resolution of 4 cm^−1^. For each EV preparation, 9 separate measurements were obtained. Between each measurement, the crystal was cleaned with methanol and background was measured against air. All ATR-FTIR spectra were subjected to automated ATR correction, automated baseline correction, and scale normalization using OMNIC 7.3 software. Scale normalization was performed over the full recorded spectral range (400–4000 cm^−1^), using identical preprocessing settings for all spectra. Spectragryph 1.2 software was used for the integration of the areas under the amide I (1725–1575 cm^−1^) and C–H stretching (2985–2840 cm^−1^) regions as previously reported [37]. These integrated values were used for relative comparison of spectral contributions between processing conditions and were not interpreted as absolute quantitative measures of protein or lipid content.

For comparison of protein-associated spectral features, mean spectra for each processing condition were examined in the Amide I/II region. For exploratory second-derivative analysis of the Amide I region, spectra were interpolated to uniform wavenumber spacing and second derivatives were calculated using a Savitzky–Golay procedure with a third-order polynomial and a 21 cm^−1^ window.

### 4.6. Flow Cytometry Analysis of Surface EV Markers

Surface EV markers were analyzed in all four preparations with latex bead flow cytometry. Latex beads (Sigma-Aldrich, St. Louis, MO, USA: 4% *w*/*v*, 4 µm) were diluted 1:10 in PBS. For each of the four EV preparations, an amount corresponding to 30 μg of Bradford-derived protein amount was immobilized onto 30 μL of diluted latex beads by overnight incubation at 4 °C with gentle rotating. Then, the beads were first blocked with 200 mM glycine (pH 7.0) for 30 min at room temperature with light rotating, followed by incubation with 5% (*w*/*v*) skimmed milk in PBS under the same conditions. The beads were then washed repeatedly with PBS after blocking until all residual milk was removed. Primary antibodies were diluted 1:5 in PBS with 1% BSA and 30 μL of antibodies was incubated with the EV-coated beads for 1 h at RT with gentle agitation. Antibodies were from Biolegend (San Diego, CA, USA): anti-CD-9 Phytoerythrin (PE)-labeled (clone MM2/57), Alexa Fluor^®^ 488 anti-human CD63 (clone H5C6) and PE/Dazzle™ 594 anti-human CD81 (TAPA-1) antibody (clone 5A6). After washing the beads with PBS, they were resuspended in 300 μL of PBS. Samples were analyzed with a BD FACS Calibur flow cytometer (BD Biosciences, San Jose, CA, USA) equipped with a 488 nm blue laser. Fluorescence emissions were detected at 525 nm (Alexa Fluor 488), 561 nm (PE), and 620 nm (PE/Dazzle). Fluorescence-positive events were determined relative to control beads that were treated in the same way but were not coated with EVs. As a negative control, Triton X-100 was added to each sample to a final concentration of 0.5% (*v*/*v*) and incubated for 30 min before analysis.

Data analysis was performed with FlowJo v10.07 software.

### 4.7. Transmission Electron Microscopy (TEM)

TEM was performed as previously described [58]. Briefly, 10 µL of the EV preparation was applied to 300-mesh Formvar-coated copper grids by grid flotation and incubated at room temperature for 45 min. The grids were then fixed with 2% formaldehyde for 10 min and washed three times (2 min each) with PBS. Post-fixation was performed with 2% glutaraldehyde for 5 min, followed by rinsing with deionized water for 5 min. The grids were air-dried at room temperature, and TEM images were acquired using a JEM-1400 transmission electron microscope (JEOL Ltd., Tokyo, Japan) operated at 120 kV.

### 4.8. Statistical Analysis

Data analysis and visualization were performed in GraphPad Prism 10.4.2 software. Differences among groups were evaluated using one-way analysis of variance (ANOVA) followed by Dunnett’s multiple comparisons test. Untreated EV preparation was used as the control group. A *p* value < 0.05 was considered statistically significant. For each processing condition, fluorescence signals in flow cytometry obtained before and after Triton X-100 treatment were compared using one-way analysis of variance (ANOVA) followed by Tukey’s multiple comparisons test. Differences were considered statistically significant at *p* < 0.05.

Principal component analysis (PCA) and calculation of mean spectra were performed in Pycharm using the NumPy v2.1.3, pandas v2.2.3., SciPy v1.16.3., Matplotlib v3.10.0, and scikit-learn v1.6.1.

Statistical analyses of repeated measurements were performed to describe differences observed at the technical measurement level. Since all processing conditions originated from a single pooled EV preparation and each treatment was applied to one aliquot, these analyses should be considered exploratory and do not represent statistical inference across independent biological replicates.

## 5. Conclusions

This study provides proof-of-concept evidence supporting ATR-FTIR as a source of orthogonal information for assessing changes in EV preparations associated with different processing conditions. Although the overall spectral profiles remained largely conserved, multivariate and targeted spectral analyses could identify the processing condition that also showed the most pronounced changes using NTA, TEM, biochemical assays and flow cytometry. The limited effects observed after repeated freeze–thawing and lyophilization, compared with the most pronounced alterations induced by combined heat and sonication, support the ability of analytical workflow to identify EV preparation subjected to the highest degree of processing stress. At the same time, the relatively modest changes observed with the milder processing conditions, both by conventional characterization methods and ATR-FTIR, indicate that the sensitivity of ATR-FTIR to more subtle alterations remains to be established. Future studies should therefore include independent EV preparations, different EV sources and isolation procedures, and a broader range of processing conditions to evaluate reproducibility, sensitivity, and broader applicability for EV quality assessment.

## Figures and Tables

**Table 1:** Nanoparticle tracking analysis (NTA) particle size distribution parameters of untreated and differently processed EV preparations.

EV Preparation	D10 (nm)	D90 (nm)	Span	<150 nm (%)	>150 nm (%)	<200 nm (%)	>200 nm (%)	150–200 nm (%)
Untreated	76.7 ± 1.0	253.0 ± 0.8	1.3	56	44	76	24	19
Lyophilized	79.5 ± 0.3	243.0 ± 2.5	1.2	59	41	78	22	19
Freeze–thawed	77.5 ± 0.7	240.1 ± 0.4	1.2	56	44	78	22	22
Heat-and-sonicated	90.1 ± 0.2	247.8 ± 5.1	1.1	49	51	74	26	26

Note: D10 and D90 represent the particle diameters below which 10% and 90% of the particle population are detected, respectively. Span represents the width of the particle size distribution. The percentages represent the proportions of detected particles within the indicated size ranges.

## Data Availability

Data can be found within the article and Supplementary Materials.

## Supplementary Materials

The following supporting information can be downloaded at: https://www.mdpi.com/article/10.3390/ijms27219482/s1.

## Abbreviations

The following abbreviations are used in this manuscript:

ATR-FTIR	Attenuated Total Reflectance Fourier-Transform Infrared Spectroscopy
DLS	Dynamic Light Scattering
EV(s)	Extracellular Vesicle(s)
MISEV	Minimal Information for Studies of Extracellular Vesicles
NTA	Nanoparticle Tracking Analysis
PCA	Principal Component Analysis
P/L	Protein-to-Lipid
SPV	Sulfo-Phospho-Vanillin
TEM	Transmission Electron Microscopy
