# Supplementary material for: Exploring FTIR Spectroscopy as an Orthogonal Approach for Detecting Processing-Induced Changes in Extracellular Vesicle Preparations
Source: Int J Mol Sci. 2026 Oct 23;27(21):9482. doi: 10.3390/ijms27219482 (PMC13762991; doi:10.3390/ijms27219482)
Supplement: Supplementary file 1 [file ijms-27-09482-s001.zip › ijms-4540745-supplementary.pdf]

EV preparation	Biochemical P/L ratio (mg/mg)	Spectroscopic P/L index (a.u./a.u.)
Untreated	7.49±2.73	0.46±0.04
Lyophilized	3.02±1.02	0.47±0.02
Freeze-thawed	3.56±1.22	0.51±0.02***
Heat-and-sonicated	2.49±0.18*	0.50±0.01**

Table S1. Comparison of biochemical protein-to-lipid ratio and spectroscopic protein-to-lipid index in untreated and processed EV preparations. The biochemical P/L ratio was calculated from Bradford-derived protein and SPV-derived lipid measurements, using BSA and cholesterol as calibration standards, respectively, and is expressed as a mass-based ratio (mg/mg). The spectroscopic P/L index was calculated as the ratio of the integrated Amide I band area to the integrated C–H stretching band area (a.u./a.u.) and therefore represents a dimensionless spectral area ratio. * Statistical analysis was performed using one-way ANOVA followed by Dunnett's multiple comparisons test. Asterisks indicate significant differences compared to the untreated group ($p < 0.05$). Statistical comparisons reflect technical measurement-level differences and should be interpreted as exploratory.

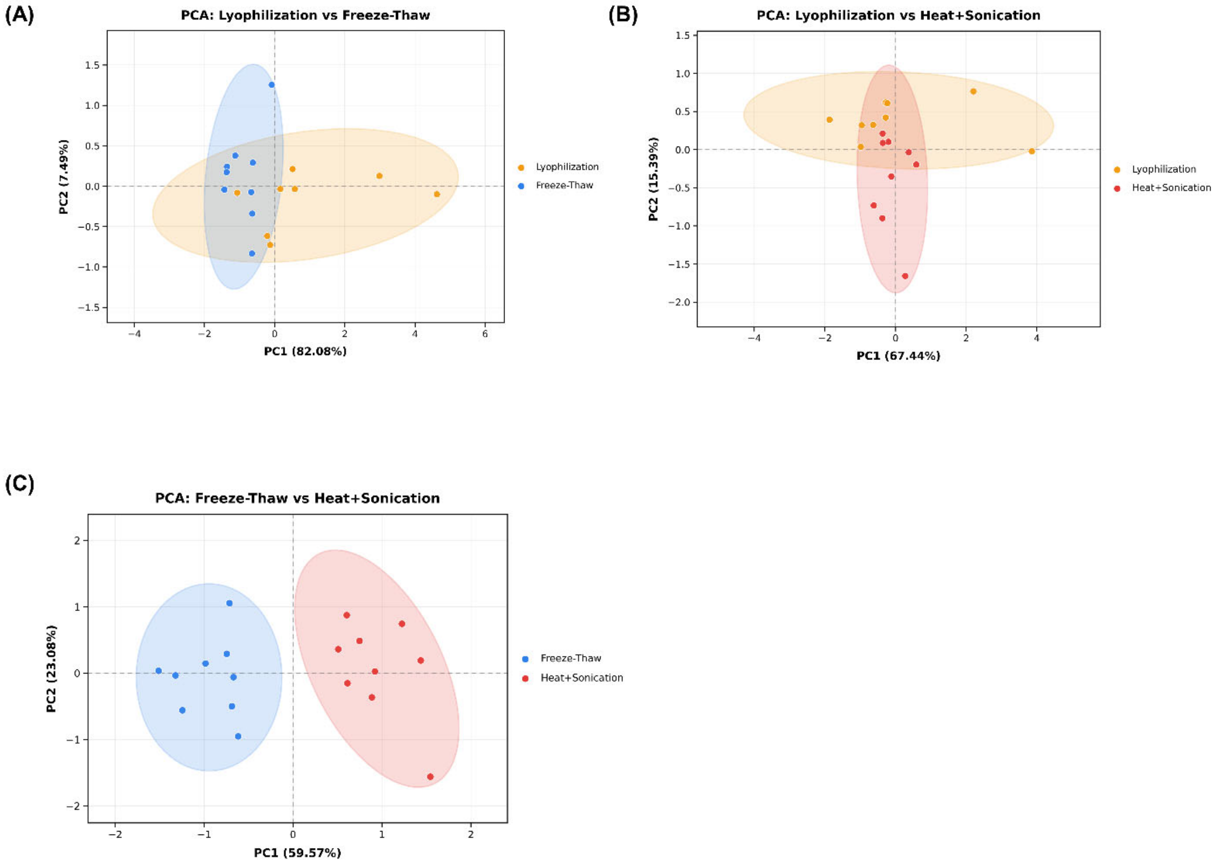

Figure S1. Pairwise principal component analysis (PCA) score plots comparing processed EV preparations. (A) Lyophilized versus freeze-thawed EV preparations. (B) Lyophilized versus heat-and-sonicated EV preparations. (C) Freeze-thawed versus heat-and-sonicated EV preparations. Each point represents one technical ATR-FTIR measurement (n = 9 per condition). Ellipses represent the 95% confidence intervals of each group. Percentages on the axes indicate the variance explained by PC1 and PC2.

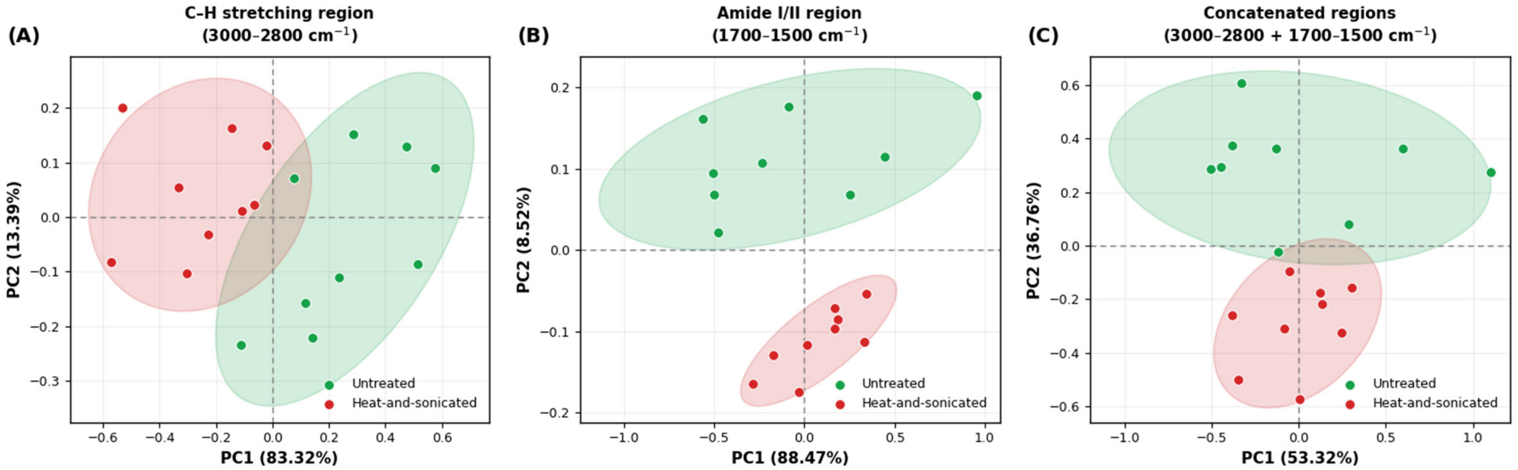

Figure S2. PCA of selected ATR-FTIR spectral regions for untreated and heat-and-sonicated EV preparations. PCA score plots were generated using (A) the C–H stretching region (3000–2800 cm^{-1}), (B) the Amide I/II region (1700–1500 cm^{-1}), and (C) the concatenation of these two spectral regions. Each point represents one technical ATR-FTIR measurement (n = 9 per condition). Ellipses indicate the distribution of scores for each group. The percentages on the axes correspond to the variance explained by PC1 and PC2.
